# Formation of Water-Free Cavity in the Process of Nafion Swelling in a Cell of Limited Volume; Effect of Polymer Fibers Unwinding

**DOI:** 10.3390/polym12122888

**Published:** 2020-12-02

**Authors:** Barry W. Ninham, Polina N. Bolotskova, Sergey V. Gudkov, Yulchi Juraev, Mariya S. Kiryanova, Valeriy A. Kozlov, Roman S. Safronenkov, Alexey V. Shkirin, Elena V. Uspenskaya, Nikolai F. Bunkin

**Affiliations:** 1 Department of Applied Mathematics, The Australian National University, Acton, ACT 2601, Australia; barry.ninham@anu.edu.au; 2 Department of Fundamental Sciences, Bauman Moscow State Technical University, 2-nd Baumanskaya str. 5, 105005 Moscow, Russia; bolotskova@inbox.ru (P.N.B.); marykrnv@bk.ru (M.S.K.); v.kozlov@hotmail.com (V.A.K.); roma-safronenkov@mail.ru (R.S.S.); 3 Prokhorov General Physics Institute of the Russian Academy of Sciences, Vavilova str. 38, 119991 Moscow, Russia; s_makariy@rambler.ru (S.V.G.); avshkirin@mephi.ru (A.V.S.); 4 Department Biophysics, Lobachevsky State University of Nizhni Novgorod, Gagarina Ave., 23, 603950 Nizhni Novgorod, Russia; 5 Department of Theoretical Physics and Quantum Electronics, Samarkand State University, University blv. 15, Samarkand City 140104, Uzbekistan; joraev-1989@inbox.ru; 6 Department of Pharmaceutical and Toxicological Chemistry, RUDN University, Miklukho-Maklaya str. 6, 117198 Moscow, Russia; uspenskaya75@mail.ru

**Keywords:** swelling of polymers, wetting angle, Nafion, Fourier transform IR spectroscopy, deuterium-depleted water

## Abstract

When Nafion swells in water, colloidal particles are repelled from the polymer surface; this effect is called the formation exclusion zone (EZ), and the EZ size amounts to several hundred microns. However, still no one has investigated the EZ formation in a cell whose dimension is close to the EZ size. It was also shown that, upon swelling in water, Nafion fibers “unwind” into the water bulk. In the case of a cell of limited volume, unwound fibers abut against the cell windows, and water is completely pushed out from the region between the polymer and the cell window, resulting in a cavity appearance. The temporal dynamics of the collapse of this cavity was studied depending on the cell size. It is shown that the cavity formation occurs due to long-range forces between polymer strands. It turned out that this scenario depends on the isotopic composition of the water, ionic additives and water pretreatment. The role of nanobubbles in the formation and collapse of the cavity were analyzed. The results obtained allowed us to conclude that the EZ formation is precisely due to the unwinding of polymer fibers into the liquid bulk.

## 1. Introduction

The polymer Nafion is intensively studied in various fields, such as physics, chemistry and hydrogen energetics (see, e.g., the lists in References [1,2,3,4,5,6] of the articles issued in 2020). In Reference [7], the histogram of publications retrieved from Web of Science from 2004 to 2014, using the keywords “Nafion” and “Nafion and fuel cell” is presented; this histogram includes several thousands of references. Basic interest derives from the use of Nafion in low-temperature hydrogen fuel cells (see, e.g., Reference [8]).

Nafion is Teflon based (substantially hydrophobic), to which hydrophilic sulfonic groups are bound [9]; Teflon is very hydrophobic, while the sulfonic group is very hydrophilic. While most work on Nafion is focused on the bulk material, the properties of water adjacent to the surface of a swollen polymer have also been explored extensively. Numerous works (see, e.g., References [10,11,12,13] and monograph by Reference [14]) describe experiments in which a Nafion membrane is immersed in an aqueous suspension of colloidal microspheres. It turned out that the microspheres are repelled from the membrane up to a distance of several hundred microns. The area from which the colloidal microspheres are effectively pushed out has been termed the “exclusion zone” (EZ). In accordance with the model developed in the monograph by Reference [14], the Nafion surface provides the adjacent layers of water a quasi-crystalline structure on a macroscopic scale; in Reference [14] (see also numerous references in this work), this effect was called the formation of the “fourth phase” of water. At the same time, it is known and generally agreed (see, for example, Reference [15] and the references therein) that a solid-state substrate can affect the properties of adjacent water only on a scale of several nanometers, i.e., the model of the peculiar phase of water looks contradictory. A recently published review [16] examines a number of alternative models for the origin of the EZ formation. In the opinion of the authors of Reference [16], the most realistic model of the EZ formation is based on the diffusiophoresis (see References [17,18,19]). Since the Nafion surface is negatively charged (see Reference [9]), a cloud of counterions near the Nafion surface is formed; the spatial distribution of the counterion density is thus non-uniform. If the ionic concentration changes significantly on a scale of the particle size, then a diffusiophoretic force begins to act on such a particle, giving rise to the motion of this particle. Since colloidal microspheres are pushed out by a distance of several hundred microns due to diffusiophoresis, it is necessary that the ion density gradients should also exist on scales of this order. However, the non-uniform distribution of ion concentration in water/aqueous electrolyte solution is realized only within the so-called Debye screening radius (see Reference [20]); for deionized water at room temperature, the Debye radius close to the charged Nafion surface is ~100 nm. Thus, it is rather questionable to explain the effect of repelling the colloid particles by a distance of several hundreds of microns due to diffusiophoresis. In this connection, it is well worth mentioning Reference [21], in which the EZ formation is attributed to a specific combination of diffusiophoresis and electrophoresis, i.e., the authors actually claim that, in an aqueous electrolyte solution, the strength of electrostatic field decays at a distance of several hundreds of microns from the charged Nafion interface. We, however, think this is a very controversial statement.

**Figure 1:**
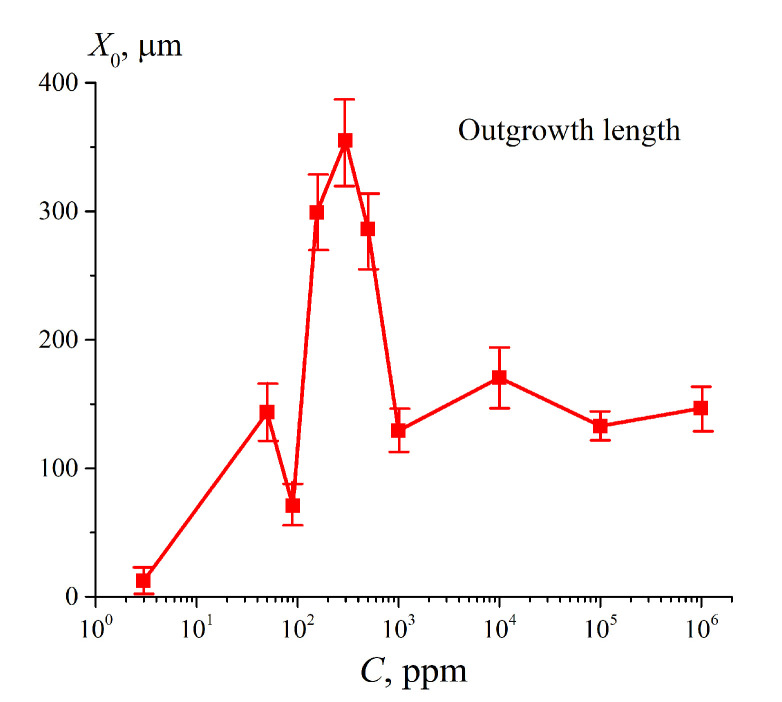
The size, *X*_0_, of the area, occupied with unwound polymer strands, vs. deuterium content of the liquid sample. See Reference [24] for more detail.

We should also mention Reference [22], in which the EZ formation was studied in suspensions of various bacteria; these bacteria were labeled with fluorescent markers. This is especially important for us, since, in our previous works [23,24], we also used the fluorescence technique; it occurred that fluorescent properties are specific for the particles of Nafion. The fluorescence intensity was studied as a function of the distance, *x*, between the Nafion surface and the pump beam in the grazing incidence geometry. In Reference [24], Nafion’s swelling was investigated depending on the deuterium content in the range from 3 ppm (the so-termed deuterium depleted water, DDW) to 10^6^ ppm (D_2_O). Note that, in ordinary (natural) water, the deuterium content is 157 ± 1 ppm (see Reference [25]).Based on the results of this experiment, the distribution of the volume number density of Nafion particles *N_naf_* (*x*) in the water bulk was found; this distribution obeys Gaussian law. It means that the particles of Nafion in the water bulk do not completely tear off the surface, i.e., we are dealing with a stationary density gradient of polymer particles in liquid. The formation of such Gaussian-like density gradient of Nafion particles was termed in Reference [24] as the “outgrowing” of Nafion fibers. In Figure 1, we exhibit the dependence obtained in Reference [24] (see Figure 9 of the work [24]) for the half-width of the Gaussian distribution *N_naf_* (*x*) (denoted as *X*_0_) vs. the deuterium content. It is seen that *X*_0_ non-monotonically varies with the deuterium content. The very first point in this graph corresponds to DDW; it is seen that the effect of “unwinding” is virtually absent for DDW.

The question arises: What will happen if the Nafion plate is swollen inside the cell with the liquid, when the distance, *L*, between the windows of the cell is less than *X*_0_? More precisely, the size, *X*_0_, should be compared with the length *l* = (*L* − *L*_0_)/2, where *L*_0_ is the thickness of the Nafion plate. If we deal with DDW, the unwinding of the polymer fibers is not revealed, *X*_0_≈0 within the experimental error, i.e., there should not be any features. However, if *X*_0_ > *L*, then we should expect that the Nafion outgrowing fibers will abut against the cell windows, and the membrane will swell under another conditions compared to those realized in the cell, whose size exceeds than *L*_0_ (e.g., a Petri dish). The present work is devoted to studying the dynamics of unwinding of polymer fibers in a constrained volume with the Fourier transform IR (FTIR) spectroscopy technique.

## 2. Materials and Methods

### 2.1. Materials

We investigated Nafion (C_7_HF_13_O_5_S·C_2_F_4_) N117 plates (Sigma Aldrich, St. Louis, MO, USA) with a thickness of *L*_0_ = 175 μm and a square area of 1 × 1 cm^2^. The Nafion plates were soaked in Milli-Q water with a resistivity of 18 MΩ⋅cm (measured immediately after the preparation) and deuterium content 157 ppm. We also studied deuterium depleted water (DDW, Sigma Aldrich, St. Louis, MO, USA), deuterium content 3 ppm. In addition, we investigated aqueous NaCl solutions prepared with Milli-Q water in the concentration range of 1–10^−14^ M, and Milli-Q water subjected to vigorous shaking with a Multi Speed Vortex MSV-3500 vibrating platform at a frequency of 20 Hz for one minute. The choice of salt concentration at the level of 10^−14^ M was motivated by the results of work [26], where the motility of *infusoria Spirostoma ambiquum* in aqueous NaCl solutions at various concentrations was studied. As shown in this work, at such NaCl concentration, the infusoria motility has a deep local minimum, approaching to almost zero. Importantly, ideas regarding the significance of substance concentration decrease and shaking were outlined among others for pharmaceutical preparations, as outlined in Reference [27].

### 2.2. Instrumentation

In this subsection, we shortly describe an experimental protocol of our FTIR experiments (for more detail, see Reference [28]). In these experiments, the transmittance spectrum *K* = *I*/*I*_0_ was measured, where *I*_0_ is the intensity of the radiation, falling on the cell with liquid and Nafion plate, and *I* is the transmitted radiation. Since the radiation in the IR range is basically absorbed by water molecules, while the Nafion does not absorb, the value of *K* should decrease upon soaking in water. In the FTIR experiments we studied the spectral range of λ = 1.8 − 2.2 μm. We have chosen this range because the absorptivity of water in this range is not so high (it is attributed to a combination of asymmetric valence and flexural vibrations of the H_2_O molecule; see Reference [29]), which allows us to register the transmitted signal behind the cell with water. The experiments were carried out on a FTIR spectrometer FSM 2201 (LLC Infraspek, St. Petersburg, Russia). The spectrometer had the following characteristics: The total spectral range is 370–7800 cm^−1^, spectral resolution is 1.0 cm^−1^ and absolute error is ± 0.05 cm^−1^. The moment, when the liquid was poured into the cell, set the reference time. Each measurement included 15 consecutive records of *K* with subsequent averaging and took 40 s (taking into account the subtraction of the background absorption due to air humidity). The time interval between each measurements was 5 min. During these intervals, the cell was removed from the spectrometer and cooled down until reaching room temperature, i.e., all measurements were carried out at the same temperature.

The experimental cell was equipped with windows made of CaF_2_, which is transparent to IR radiation in the spectral range under study. The quality of polishing the windows was important: The size of the roughness was 2.5–5 μm. Before each experiment, the windows were rinsed with Milli-Q water and then dried by a stream of chemically pure nitrogen. The distance, *L*, between the windows was varied from 180 to 220 μm, with a step of 10 μm.

## 3. Results and Discussion

### 3.1. Formation of an Empty Cavity inside the Cell

**Figure 2:**
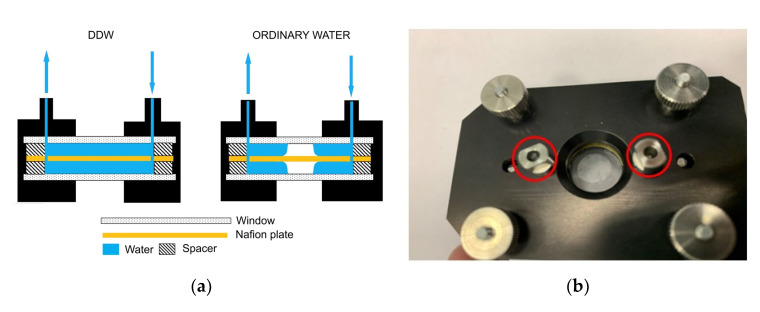

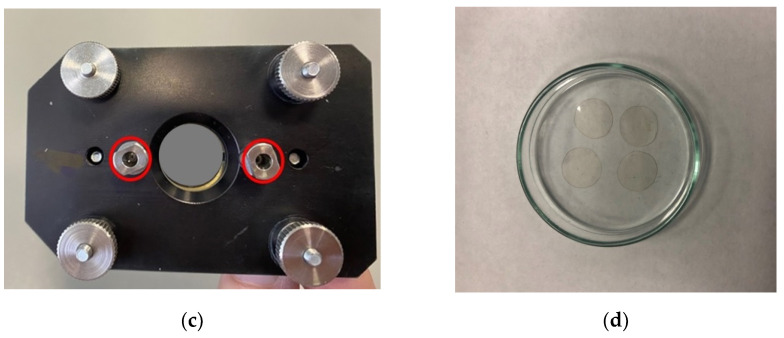
The cell used in FTIR spectrometry experiments. (**a**) Schematic drawing of the cell design in the case of filling with deuterium depleted water (DDW) and ordinary water. (**b**) Photo of the cell immediately after filling with ordinary water. The inlet/outlet holes are highlighted in red. (**c**) Photo of the cell immediately after filling with DDW. The inlet/outlet holes are highlighted in red. (**d**) Photo of the Nafion plates used in FTIR experiments.

In Figure 2a–d, we exhibit the cell with Nafion plate and water. Figure 2a illustrates, schematically, a spontaneous formation of an empty cavity, when the cell is filled with ordinary water, and the absence of the cavity when filled with DDW. Figure 2b shows a photograph of the cell immediately after filling with ordinary water; an empty cavity is clearly seen. Figure 2c shows a photograph of the cell immediately after filling with DDW; in this case, the cavity is absent. It was very important that the volume of the liquid poured into the cell always exceeds the volume of the cell, i.e., if we pour a liquid into the inlets (marked on Figure 2a with arrow directed downwards, and on Figure 2b,c in red), then water should spill out of the outlet (marked on Figure 2a with arrow directed upwards). With this protocol of filling, gas bubbles do not form inside the liquid, and the entire volume of the cell (except for the cavity) is filled with water. After filling, the inlet and outlet holes were closed with Teflon inserts, but not very tightly, i.e., there was a possibility of air to access into the cell.

According to our qualitative model, the cavity is formed due to an instantaneous unwinding of polymer fibers into the liquid bulk, followed by touching the cell windows. This gives rise to local shear stresses, which stimulate “squeezing” of water molecules from the areas between the hydrophobic strands. Since the polymer fibers forcefully abut against the cell windows, we can speak about an “enhancement” of the hydrophobic effect: Due to local stresses, the water molecules are pushed out very efficiently from the areas between the fibers. At the same time, the peripheral areas of the plate (see Figure 2a,b) are always in contact with water, and the polymer fibers in these areas keep swelling, which results in eventual collapse of the cavity. It is clear that the rate of the collapse should depend on the size of the Nafion plate area covered with water. Therefore, Nafion’s plates were prepared in such a way that they had approximately the same size and shape (see Figure 2d).

It is very important that the cavity is absent in the case when filling with DDW. The cavity absence in this case can be explained by the fact that for DDW there is no effect of the polymer fibers outgrowth (see Figure 1). Thus, we can claim that the formation of the cavity is due to outgrowing the polymer strands. In Figure 2, we present the photos for the cell with a distance between the windows *L* = 200 μm. Bearing in mind that the thickness of the Nafion plate is *L*_0_ = 175 μm, we find that in this case an area of size *l* = (*L* − *L*_0_)/2 = 12.5 μm is occupied by unwound polymer fibers from each side of the Nafion plate.

### 3.2. Transition from the Hydrophobic to the Hydrophilic State

**Figure 3:**
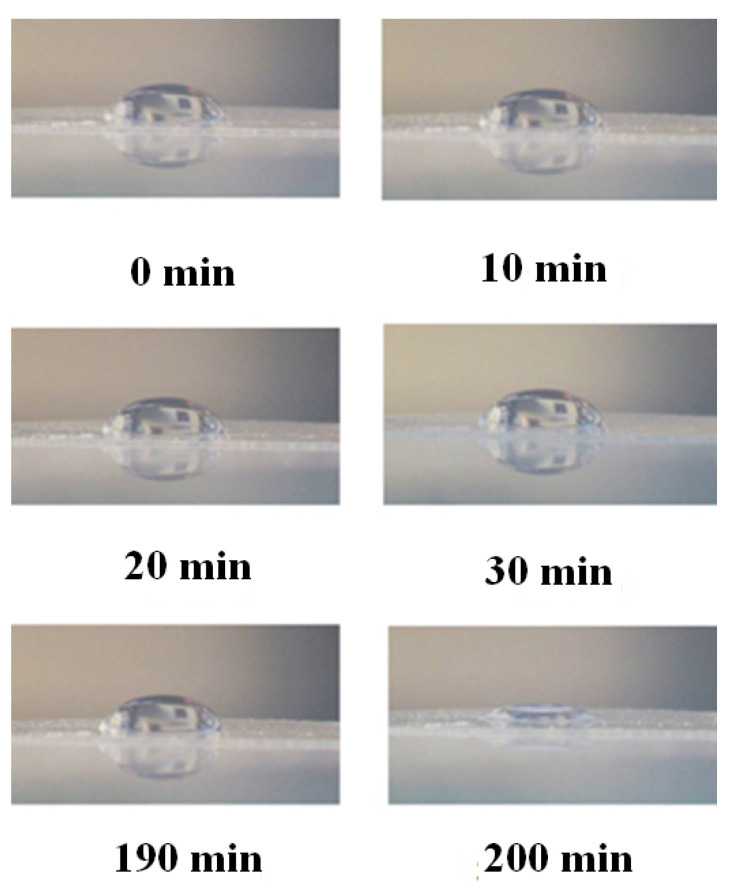
The change of wetting angle of a water droplet on the surface of Nafion membrane vs. the time of soaking the membrane in water. The photo was taken by one of authors.

As is known [9], the Nafion initially has hydrophobic properties, i.e., the wetting angle for a drop of water on the membrane surface is ~90°. Upon swelling, the membrane becomes hydrophilic, and the wetting angle becomes substantially less than 90 degrees. In Figure 3, we show photographs that illustrate the dynamics of the transition from hydrophobic to hydrophilic state. In this experiment we put a droplet of water of a fixed volume on a surface of dry Nafion plate, mounted on a smooth horizontal substrate; this moment corresponds to the time reference, and the change of wetting angle was monitored visually. Then the plate was placed in a Petri dish with ordinary water for 10 min, and after removal the plate from the Petri dish a water droplet was once again put on the surface. As is seen, the wetting angle is constant for 190 min, but at 200-th min the wetting angle sharply decreases, i.e., Nafion becomes hydrophilic. As shown in Reference [24], the unwinding of polymer fibers into the bulk water occurs immediately after immersing in water. Thus, the transition to the hydrophilic state, illustrated in Figure 3, occurs essentially under the conditions when the polymer fibers are already unwound towards the water bulk. Obviously, in the experiment, illustrated in Figure 3, the unwinding effect is not in any way manifested.

### 3.3. Transmission Spectrum in FTIR Experiments

**Figure 4:**
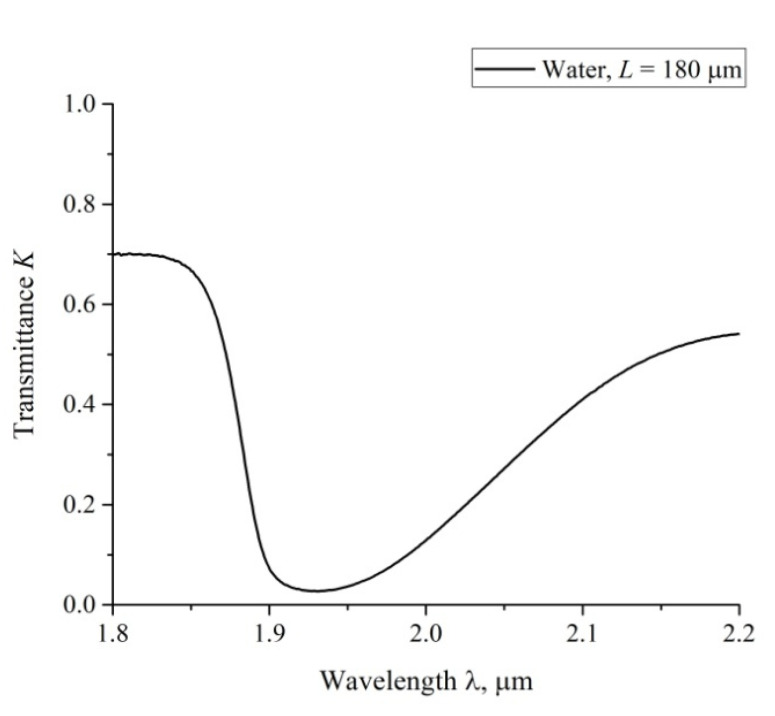
The spectrum of transmittance, *K*, for water in the range 1.8 < λ < 2.2 μm; the distance between the cell windows *L* = 180 μm.

According to the Lambert–Bouguer–Beer law (see, e.g., Reference [30]), the transmitted beam intensity, *I*, meets formula $I=I_{0}\;\exp\left(-\kappa L\right)$, where *κ* is the extinction coefficient, *L* is the distance between the cell windows. In Figure 4 we show a typical example of a transmittance spectrum *K* for ordinary water, poured into a cell with *L* = 180 μm. For the spectral range 1.8 < λ < 2.2 μm the spectral minimum *K*_min_ is related to λ = 1.93 μm. For short swelling times *t* and small distances *L* we have *K* (λ = 1.8 μm) ≈ 0.7. Upon increasing *t* and *L*, the value of *K* (λ = 1.8 μm) slightly decreases, while *K* (λ = 2.2 μm) decreases more strongly. The decrease in *K* at the wavelengths λ = 1.8 and 2.2 μm is basically due to the contribution from a more intense absorption band centered at λ = 3 μm. Since we are interested in the quantity |ln *K*_min_| at the wavelength λ = 1.93 μm, it makes sense to count the value of *K*_min_ from the level, which is the same to all spectrograms. Indeed, since |ln *K*_min_| at *K*_min_ < 1 is very steep function, the inaccuracies in finding *K*_min_ should result in large errors of |ln *K*_min_|. This is why we put herein below *K* (λ = 1.8 μm) = 0.7 for all spectra.

**Figure 5:**
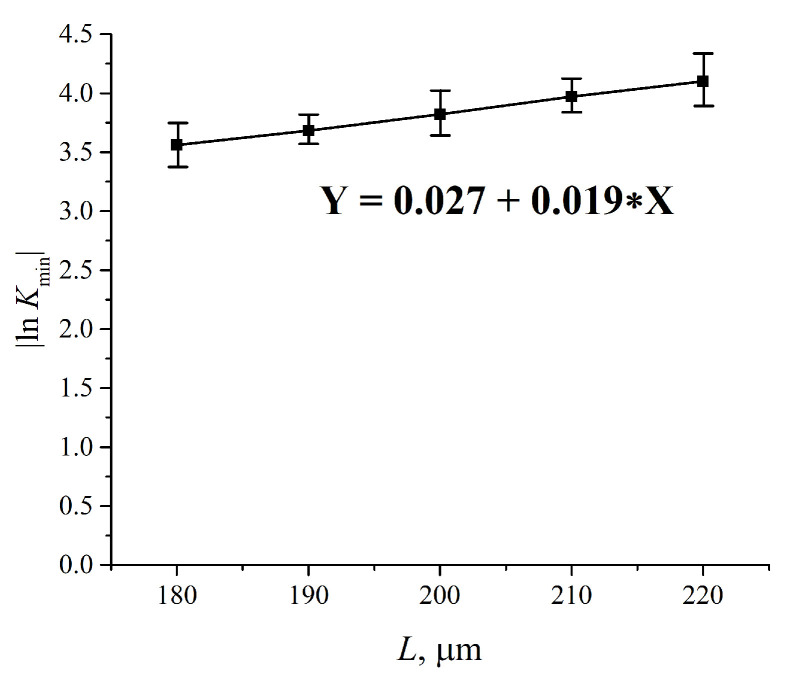
Dependence |ln *K*_min_| vs. the distance, *L*, between the cell windows for water. *K*_min_ is related to the spectral minimum of the graph in Figure 4. The dependence |ln *K*_min_| (*L*) is well approximated by function Y = 0.027 + 0.019 X.

In Figure 5, we present the results of measuring |ln *K*_min_| for water vs. the distance, *L*, between the windows; *L* = 180, 190, 200, 210 and 220 μm. The experimental dependences are the result of averaging over five consecutive measurements. The choice of the minimum value *L* = 180 μm is explained by that the thickness of the Nafion plate *L*_0_ = 175 μm; the choice of the maximum value *L* = 220 μm is due to the fact that in this case the intensity *I* of the transmitted beam approaches zero, i.e., the results of measurements become incorrect. The dependence |ln *K*_min_| vs. *L* is well approximated by a linear function *Y* = 0.027 + 0.019 *X*, i.e., we obtain for the extinction coefficient *κ* ≈ 0.019 μm^−1^. The value of |ln *K*_min_| for dry Nafion was also measured. In this case, the absorption is due to water molecules encapsulated inside the nanometer-sized cavities in the polymer matrix (see Reference [9]), i.e., |ln *K*_min_|= *κ* (*C_w_*)_0_*L*_0_, where (*C_w_*)_0_ is the concentration of water in dry Nafion; we obtain (*C_w_*)_0_ = 0.174.

**Figure 6:**
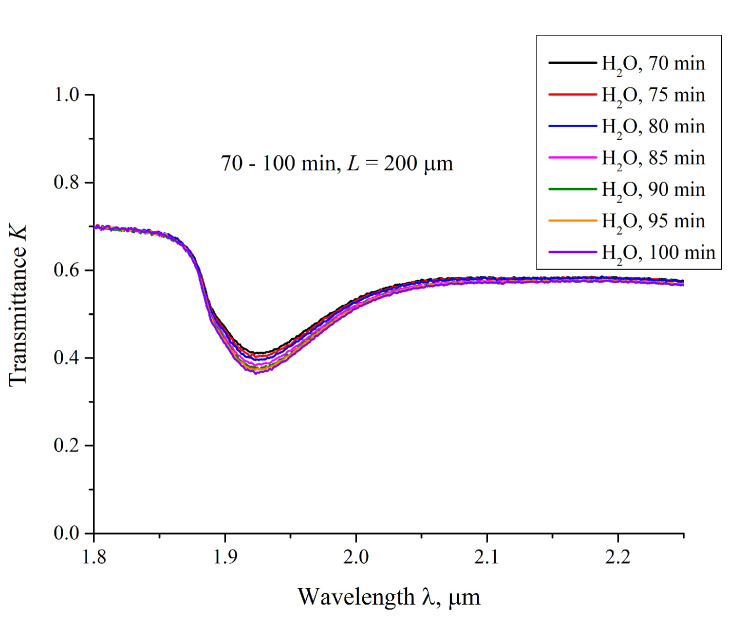
The transmittance spectra in the range 1.8 < λ < 2.2 μm for the case of swelling Nafion in ordinary water; the distance between the windows is *L* = 200 μm, and the curves are related to the swelling times 70, 75, 80, 85, 90, 95 and 100 min.

We also measured the transmittance spectra *K* for Nafion, swelling in water, with an interval of 5 min. In Figure 6 we present typical spectra, taken for 70 < *t* < 100 min for ordinary water; the distance *L* = 200 μm. It is seen that *K* decreases smoothly upon soaking.

**Figure 7:**
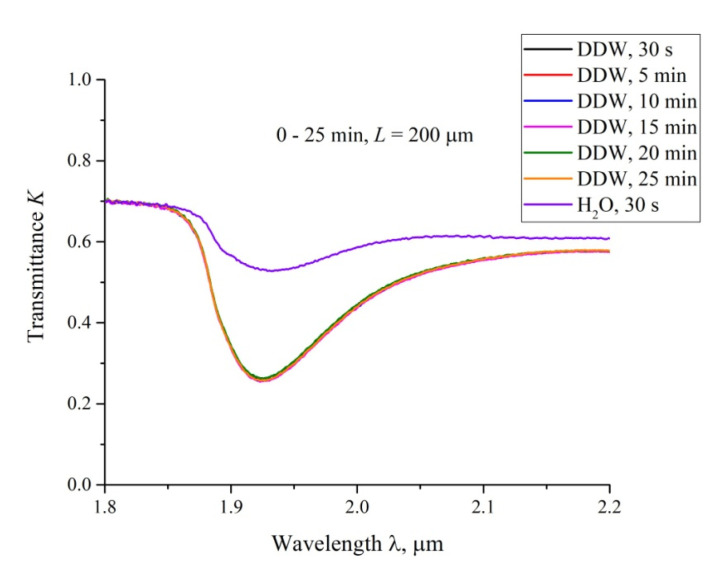
The transmittance spectra in the range 1.8 < λ < 2.2 μm for DDW for the distance between the windows, *L* = 200 μm. The graphs are related to the times of swelling 0.5, 5, 10, 15, 20 and 25 min. The upper curve is related to the transmittance spectrum for ordinary water; the time of swelling is 0.5 min.

In Figure 7, we exhibit the transmittance spectra for DDW; *L* = 200 μm, and the swelling times 0 < *t* < 25 min. Here, *t* = 0 corresponds to the moment of filling the cell with liquid and conducting the very first measurement, which takes about 15 s, i.e., the first graph corresponds to the time *t* ≈ 30 s after filling. It is seen that the spectra for DDW are almost identical for all swelling times. We also present the spectrum of *K* for ordinary water, taken at *t* ≈ 30 s after filling the cell. It can be seen that this spectrum differs significantly from the DDW spectra. According to Reference [28], the transmission spectra of water in the range 1.8 < λ < 2.2 μm are the same for the samples with the deuterium content 1−10^4^ ppm, i.e., the detected effect cannot be associated with various absorptivity of ordinary water and DDW. We associate the difference in these spectra with the absence of the empty cavity in DDW.

**Figure 8:**
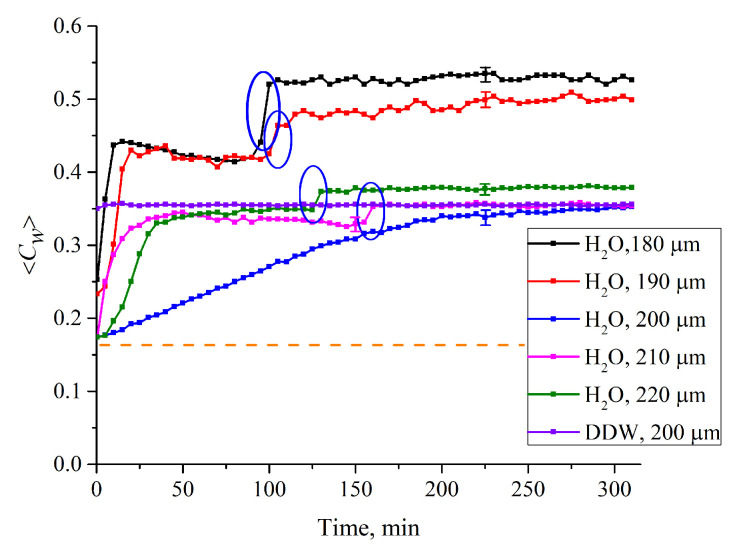
Average (over the distance, *L*, between the windows of the cell) concentration of water 〈*C_w_*(*t*)〉 vs. the time of swelling for various *L* for ordinary water. The graphs are related to the distances *L* = 180, 190, 200, 210 and 220 μm; we also show a graph for DDW for *L* = 200 μm. The ellipses mark “second jumps” in the dependences 〈*C_w_*(*t*)〉 (see the comments in the text). The dashed line is water concentration for dry Nafion (baseline).

Based on the spectra obtained, we can find the dependence of water concentration 〈*C_w_*(*t*)〉 vs. the swelling time, *t*, averaged over the length *L*. We rewrite the Beer–Lambert law as follows:

(1) $$I\left(t\right)=I_{0}\exp\left(-\kappa \overset{L}{\underset{0}{\int }}C_{w}\left(t,x\right)dx\right)\approx I_{0}\exp\left(-\kappa \langle C_{w}\left(t\right)\rangle L\right),$$

where we get for water concentration in the spectral minimum $\langle C_{w}\left(t\right)\rangle =\frac{\left|lnK_{min}\left(t\right)\right|}{\kappa L}$. Figure 8 shows the dependences 〈*C_w_*(*t*)〉 for the cells with *L* = 180, 190, 200, 210 and 220 μm. The dashed line is related to te concentration of water in dry Nafion (*C_w_*)_0_ = 0.174 (baseline).

Let us first consider the dependence 〈*C_w_*(*t*)〉 for *L* = 180 μm. As is seen, 〈*C_w_*(*t*)〉 demonstrates a sharp jump after ~10 min of swelling, which is accompanied by a collapse of the cavity. We will call it the “first jump”. Further, at *t*~90 min of swelling, another jump occurs, (highlighted by a blue contour), which is not associated with the cavity collapse; we will call this the “second jump”. Note that the second jumps, only with a smaller amplitude, are also observed for *L* = 190, 210 and 220 μm (highlighted by the corresponding contours).

**Figure 9:**
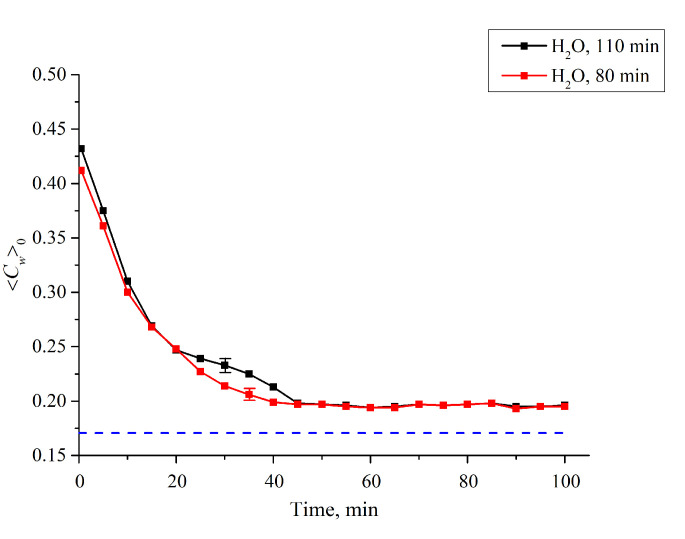
Average (over the Nafion plate thickness *L*_0_ = 175 μm) water concentration 〈*C_w_*(*t*)〉_0_ inside the membrane vs. the time of drying after soaking within 80 and 110 min in the cell with the distance between the windows as *L* = 180 μm. It is seen that the dynamics of drying are different for the times 80 and 110 min. The dashed line is the water concentration for dry Nafion (baseline).

Since the first jump is associated with the cavity collapse, after the completion of this jump the entire polymer surface is covered with water, which is related to the common conditions of the polymer swelling. Thus, the second jump should be associated with a sharp increase of 〈*C_w_*(*t*)〉 in the membrane bulk. To test this hypothesis, we studied the temporal behavior of water concentration in the membrane bulk $\langle C_{w}{(t)\rangle }_{0}=\frac{\left|lnK_{min}\left(t\right)\right|}{\kappa L_{0}}$ upon drying the membrane; here, *L*_0_ = 175 μm is the membrane thickness. In this experiment, the membrane was immersed in the cell with *L* = 180 μm (for definiteness), and then soaked for *t*_1_ = 80 and *t*_2_ = 110 min, i.e., before and after the second jump. Then the membrane was removed from the liquid and subjected to drying, in air, at room temperature (see Figure 9). As is seen, the dependences 〈*C_w_*(*t*)〉_0_ vs. the time of drying for the times *t*_1_ and *t*_2_ are different. This indirectly confirms that the second jump in the dependence 〈*C_w_*(*t*)〉 in the interval *t*_1_ < *t* < *t*_2_ (Figure 8) is associated with a sharp increase of the water content inside the membrane (see, e.g., Reference [31] for more detail).

Let us return once again to Figure 3, where the Nafion plate was soaked in ordinary water in a Petri dish (the liquid volume significantly exceeds the volume of the Nafion plate). Obviously, during such soaking, polymer fibers unwind towards the water bulk at a distance *X*_0_ ≈ 300 μm (see Figure 1), and the transition to the hydrophilic state is completed within the interval 190 < *t* < 200 min. It is clear that for the same Nafion plate, which swells in ordinary water in a cell with *L* = 180 μm, the outgrowing effect is essentially suppressed due to the geometric factor. In our opinion, in this case, the transition to the hydrophilic state should happen earlier than in the case of swelling in a Petri dish. This hypothesis is supported by the 〈*C_w_*(*t*)〉 dependences for the cells with thicknesses *L* = 190, 210 and 220 μm. For these cells, the second jump also occurs earlier than the transition to the hydrophilic state at swelling in the Petri dish. Thus, we can claim that the outgrowth of polymer fibers slows down the transition to the hydrophilic state. At the same time, as is seen in Figure 8, for DDW there are no features like the first and second jumps.

In Figure 8 we show the dependence 〈*C_w_*(*t*)〉 for ordinary water for *L* = 200 μm; in this case the empty cavity arises (as for other distances *L*), but does not completely disappear for ~ 200 min. The question arises of why the cavity collapses so slowly in the case where the area, which can be occupied with unwound fibers, has the size *l* = (*L* − *L*_0_)/2 = 12.5 μm from each side of the membrane? We still do not know why this size is so specific for the Nafion fibers in water. We only can assume that this size is exactly a multiple of a certain characteristic scale *l*’ of Nafion’s fibers (i.e., 12.5 μm = *nl*’, where *n* is an integer), which provides an additional “stiffness” to these fibers when they unwound normally to the cell windows; this should slow down the cavity collapse.

**Figure 10:**
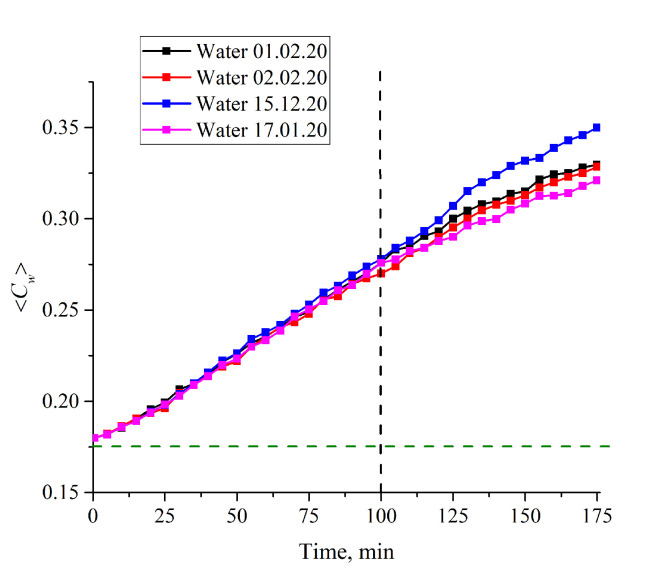
Average (over the distance *L* = 200 μm between the windows of the cell) concentration of water 〈*C_w_*(*t*)〉 vs. the time of swelling for ordinary water received on different days from various Milli-Q plants. It is seen that the graphs actually coincide to one another within the first 100 min of swelling. The horizontal dashed line is water concentration for dry Nafion (baseline).

Let us consider now the case of *L* = 200 μm in more detail. In Figure 10 we exhibit the dependences 〈*C_w_*(*t*)〉 for water, received at different Milli-Q setups on different days. Obviously, it makes no sense to calculate experimental errors within the first 100 min of swelling: The points on the graphs actually coincide, and only at times *t* > 100 min does the spread between the extreme points along the ordinate axis (the scatter in the dependence 〈*C_w_*(*t*)〉) exceed 1%. This is why, in Figure 8, we present the experimental errors only for *t* > 100 min; the values of these errors were calculated based on the data of five consecutive measurements.

### 3.4. Contribution of Gas Nanobubbles in the Cavity Formation/Collapse

#### 3.4.1. Possible Role of Roughness of the Windows

**Figure 11:**
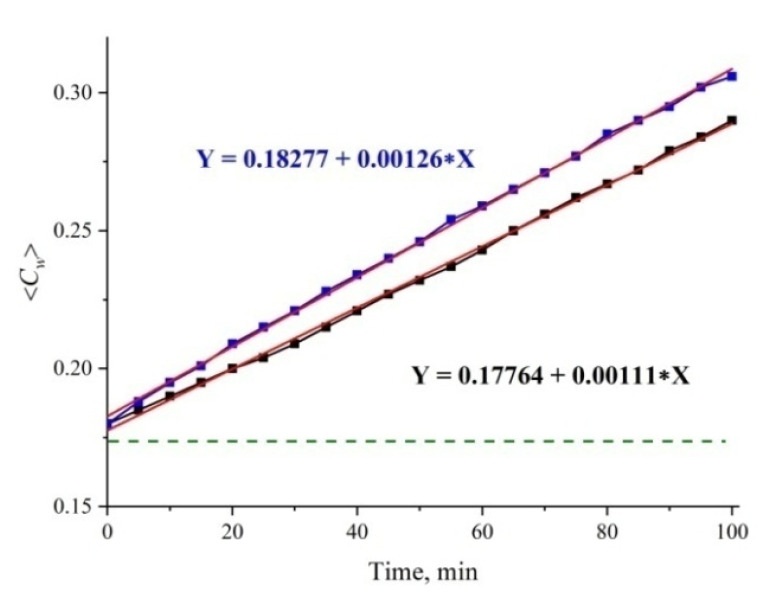
Average (over the distance *L* = 200 μm between the windows of the cell) concentration of water 〈*C_w_*(*t*)〉 vs. the time of swelling for two cells with different roughness of the windows. The upper line is approximated by function Y = 0.18277 + 0.00126 X; the lower line is approximated by function Y = 0.17764 + 0.00111 X. The dashed line is the water concentration for dry Nafion (baseline).

A further analysis will be devoted to the study of possible effects of gas nanobubbles in the cavity collapse. Indeed, the collapse rate can depend on the degree of roughness due to the well-known fact that nanometer-sized gas bubbles appear on the tips and rough segments of a solid substrate (see, for example, Reference [32] and the references therein). Since the outgrown Nafion fibers abut against the window surface, the rate of the cavity collapse should be controlled by a specific interaction between the unwound fibers and the surface of the cell window (we conditionally call this interaction “friction force”). In Figure 11 we exhibit typical examples of dependences 〈*C_w_*(*t*)〉 for the first 100 min of swelling for ordinary water and two different cells; it is seen that the straight line dependence is retained for both cells, but for one cell the slope coefficient is *k* = 0.00126, while for the other one *k* = 0.00111. Since the cell windows were purchased from the same manufacturer and were not subjected to any special processing, the only explanation for the unequal slopes is a different degree of surface roughness of the windows. Intuition suggests that for rougher surfaces, the rate of the cavity collapse should be less, i.e., for these windows *k* = 0.00111; all the results in this paper are related to the cell with *k* = 0.00111.

For a more detailed analysis, it is necessary to carry out experiments in which the window surface will be modified by imparting them hydrophobic /hydrophilic properties. In our previous work [33], we specially explored the density of nanobubbles near hydrophobic and hydrophilic surfaces in experiments with optical cavitation in water and aqueous salt solutions. It turned out that the density of nanobubbles is substantially higher close to the hydrophobic surface as compared to the hydrophilic one.

#### 3.4.2. The Role of Ionic Additives

**Figure 12:**
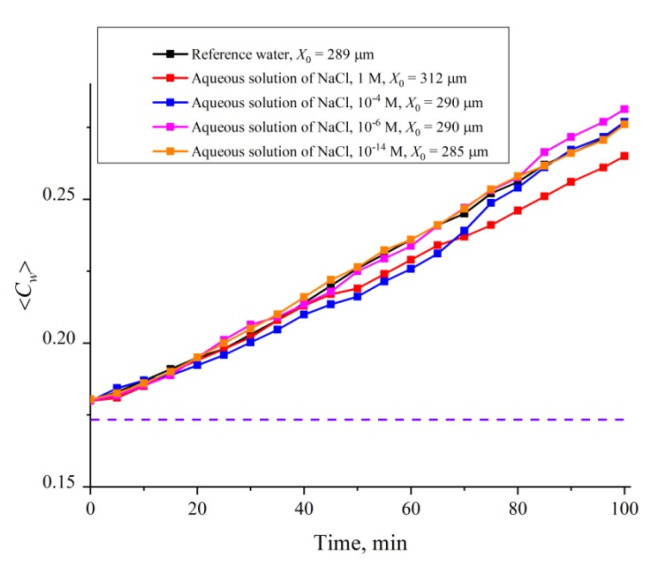
Average (over the distance *L* = 200 μm between the windows of the cell) concentration of water 〈*C_w_*(*t*)〉 vs. the time of swelling for aqueous NaCl solution with concentrations of 1, 10^−4^, 10^−6^ and 10^−14^ M. The estimates of *X*_0_ (see Reference [24] for more detail) are also given. The dashed line is water concentration for dry Nafion.

It turned out that the dynamics of the cavity collapse is controlled not only by the roughness of the cell windows, but also by the ionic additives. In Figure 12 we show a set of dependences of 〈*C_w_*(*t*)〉 for an aqueous NaCl solution for 0 < *t* < 100 min. The ionic concentration was varied in the range 10^−14^–1 M; the dependence for reference ordinary water is also given in Figure 12. These investigations are also devoted to the possible contribution of nanobubbles to the cavity collapse. As shown in References [34,35], ionic additives (similar to local roughness on the cell windows) should result in increasing the volume number density of nanobubbles. In order not to clutter up the graph, we present in Figure 12 the data only for concentrations of 1, 10^−4^, 10^−6^ and 10^−14^ M. We also present estimates of the size *X*_0_ of the area occupied by polymer fibers when they are unwound into a cell of large size, exceeding essentially the Nafion plate size (a Petri dish); these experiments were carried out in accordance with the protocol described in Reference [24]. As follows from the dependences obtained, the size of *X*_0_ increases with growing the ionic concentration, but at a concentration of ≤10^−4^ M, the value of *X*_0_ reaches the level of the reference water. At the same time, the run of the dependences 〈*C_w_*(*t*)〉 are not the straight line up to a concentration 10^−14^ M; only for this concentration the dependence in the entire range 0 < *t* < 100 min is rectilinear and practically coincides with the dependence for the reference water. Thus, deviations from the rectilinear dependence can be associated in this case with the addition of foreign ionic impurities.

#### 3.4.3. The Role of Shaking

**Figure 13:**
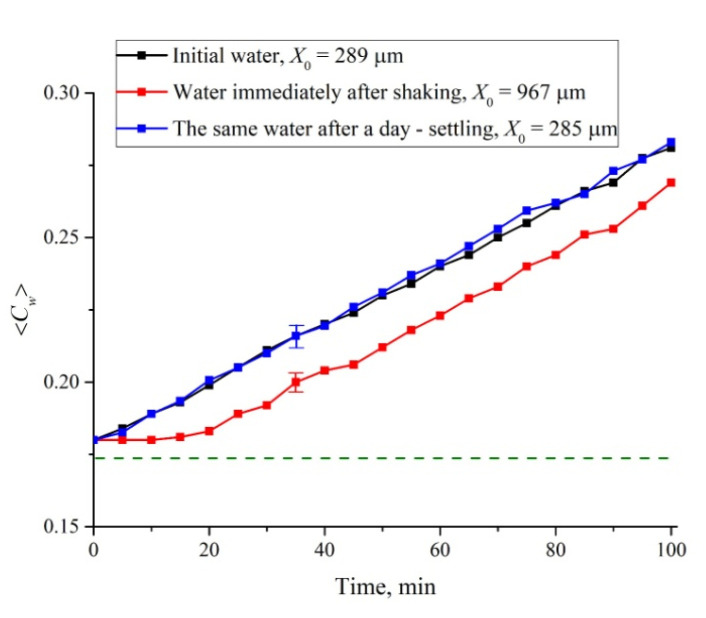
Average (over the distance *L* = 200 μm between the windows of the cell) concentration of water 〈*C_w_*(*t*)〉 vs. the time of swelling for initial water, water subjected to shaking, and water that settled for one day after shaking. The estimates of *X*_0_ (see Reference [24] for more detail) are also given. The dashed line is the water concentration for dry Nafion.

Finally, we studied water samples subjected to intensive shaking on a Vortex MSV-3500 vibrating platform at a frequency of 20 Hz for one minute. These studies were motivated by the results of References [36,37], where it was shown that shaking leads to an increase in the volume number density of nanobubbles by about 10 times. However, after shaking and settling for a certain time, the original properties of water should be restored. In Figure 13 we exhibit the dependence of 〈*C_w_*(*t*)〉 for the swelling time 0 < *t* < 100 min before/immediately after shaking, and after a day of settling. In addition, we present the estimates of the size *X*_0_. Immediately after shaking and for the first 15 min, 〈*C_w_*(*t*)〉 growth is clearly slowed down compared to the initial water. It is also seen that the size *X*_0_ for shaken water is about three times higher compare to initial water. However, after a day of settling, the liquid returns to its original state: The rectilinear dependence and the value *X*_0_ are restored. Summarizing, we can claim that the presence of nanobubbles slows down the cavity collapse.

## 4. Discussion

### 4.1. The Similarities between the EZ and the Glycocalyx in Physiology

As shown above, the cavity formation is due to the unwinding of initially hydrophobic polymer fibers into the liquid bulk. The first question we pose as follows: Is it possible for a strand of Nafion polymer to unwind to the width of the EZ? The answer is yes and comes from biology. For example, the DNA molecule unwinds and rewinds to pack inside the cell nucleus with extravagant ease and reproducibility, while the length of a single macromolecule can amount to a meter (see, e.g., Reference [38]). Thus, it is highly possible to have densely packed polymeric strands at the Nafion surface that can unwind to span the enormous width. It is also possible for such stretched-out strands to form a rigid parallel palisade, which can repel colloidal microparticles; this constitutes the effect of the exclusion zone.

We should remind here a curious phenomenon in biology that does seem to provide insights into the EZ formation. For living cells there exists, at their surfaces, the Endothelial Surface Layer (ESL) (see, e.g., the monograph by Reference [39]). In our opinion, ESL is an analogue of the EZ of Nafion in the sense that ESL repels colloidal particles like red blood cells and bacteria and over micron distances (see, e.g., Reference [40]). The substrate on which the ESL sits is a tangled layer of predominately charged hydrocarbon polymers parallel to the venous cell surface. It consists of 50–90% heparan sulfate, and the remaining 10–50% is dermatan sulfate, chondroitin sulfate, keratan sulfate and hyaluronan (see, e.g., Reference [41]). Thus the difference to Nafion is that the glycocalyx is made of hydrocarbon polymers plus sulfate, not fluorocarbons plus sulphonate as for Nafion; note that the fluorocarbon group of Nafion is much more hydrophobic than the hydrocarbon groups of the ESL. The width of the endothelial glycocalyx (the “biological EZ”) is about 500 nm. This is an enormous distance compared with the thickness of the cell lipid membrane (which is ~2 nm), on which the glycocalyx sits.

**Figure 14:**
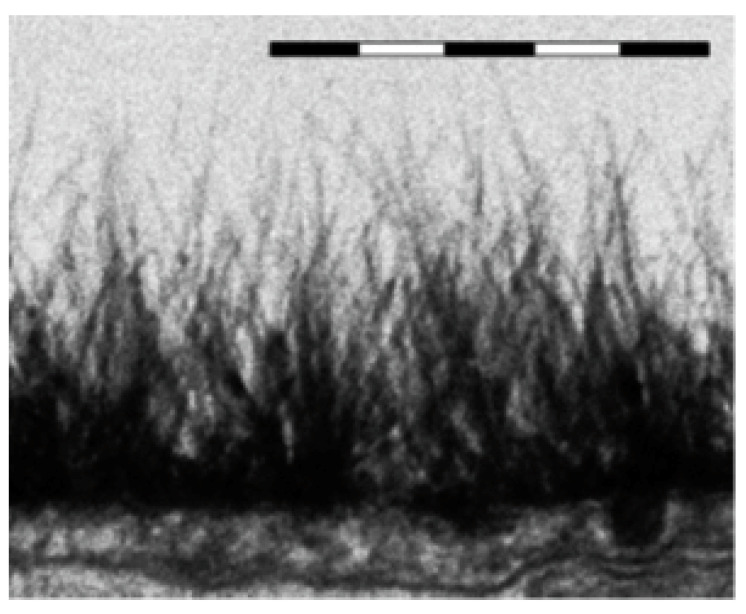
Electron microscopy image of the endothelial glycocalyx in rat myocardial capillary; reprinted from Figure 1c in Reference [42]. Bar above image is 0.5 μm. Reprinted with permission from H. Vink et al., Copyright 2003 AHA Journals.

There are differences between ESL and EZ: The ESL exists and is bounded by the bloodstream, which is at a physiological salt concentration that is equivalent to 0.14 M for NaCl solution. It is fed and replenished by carbon dioxide nanobubbles from metabolism emerging from within the tissue (see Reference [40] for more detail). The result is a dynamic foam bounded by dilute polymeric strings perpendicular to the surface; see Figure 14, where electron microscopy image of the endothelial glycocalyx in rat myocardial capillary is given (reprinted from Figure 1c in Reference [42]). We emphasize, once again, that ESL has the EZ property, repelling colloidal particles. In this case the colloidal particles are bacteria, T-cells, red blood cells and so on, i.e., it is essentially the same phenomenon as for Nafion, but in lower key.

### 4.2. Very Long-Range Forces

With these facts established, we go on to discuss some other components of the EZ formation. If we keep in mind the idea of stretched parallel polymeric strands for the EZ structure, there exist forces acting to maintain the spatial structure of these strands (see Reference [15] and the references therein). These forces are specific for charged thin strands (cylinders) of Nafion. Their charge is due to the hydronium counterions of Nafion (see Reference [9] for more detail). The attractive forces arise due to correlations in charge fluctuations, and have a dispersion (high frequency) component that varies as *V*(*R*)~(1/*R*)^2^[ln(*R*/*a*)^3/2^], where *R* is the distance between parallel cylinders per unit length and *a* is the width of the cylinders (for more detail, see References [43,44]). In accordance with the theoretic model outlined in References [43,44], we deal with non-additive very long range forces, wherein the attractive cooperative many body forces are opposed by the repulsive electrostatic double layer forces between the thin cylinders. Summarizing, we have a sparsely packed layer of anisotropic parallel polymer strands at the surface. These are aligned perpendicular to the solid Nafion surface. This structure is stabilized by very strong and cooperative forces. As was shown in Reference [23], the spatial structure of the Nafion strands close to the polymer–water interface can be considered as a special type of photonic crystal, which was supported by the data of the birefringence experiments. Currently, we are developing a quantitative theory of the spatial interaction of polymer fibers unwound in the water bulk, based on the results presented in References [43,44].

Continuing to develop the analogy between Nafion and the cell membrane, it should be noted that the coating of enzymatic electrochemical sensors with a Nafion layer leads to an increase in the performance of such sensors (see, for example, Reference [45], where a bioelectrochemical sensor based on PQQ-dependent glucose dehydrogenase in the glassy carbon electrode, coated with Nafion layer and over-coated by an enzyme layer, crosslinked by glutaraldehyde, has been described). The results obtained in Reference [45] can also be explained by the effect of outgrowing the polymer fibers and the retention of water in the structure of the unwound fibers. Thus, the enzyme PQQ-dependent glucose dehydrogenase described in Reference [45], immobilized within the Nafion layer, has greater catalytic activity with Nafion layer, which is crucial for the preservation of the native structure of the enzyme.

The question arises as to what the specificity of the size *l* = (*L* – *L*_0_)/2 = 12.5 μm is when Nafion swells in ordinary water; see the graph in Figure 8 for the cell with the distance between the windows *L* = 200 μm. We do not yet have a clear answer to this question. We can only assume that the size *l* = 12.5 μm is a multiple of the length of a certain segment *l*’ of unwound polymer fibers, i.e., *l* = *nl*’, where *n* is an integer. Since the Nafion surface is negative, the unwound polymer fibers are also negative, and due to Coulomb repulsion from the membrane surface these fibers are directed perpendicular to the membrane surface; this is directly confirmed by the calculations based on the results of the studies by References [43,44] (see also our prior work, Reference [23]). If the length of the polymer fiber can be expressed as *nl*’, where *N* is the total number of segments of length *l’*(*N* > *n*), then for these *n* segments the polymer fiber should be directed exactly perpendicular to the window surface, as was shown in Reference [23]; the orientation of the remaining *N—n* segments is not important for us. Obviously, in this case, the rigidity of the polymer fibers, which rested against the cell window, should be maximal. At the same time, if the condition *l* = *nl*’ is not met, then the fibers experience bending, and the stiffness of the fibers should be significantly less. Since the rate of the cavity collapse is controlled by the “friction force” between the window surface and the fibers, and the friction force is determined by the magnitude of the normal pressure on the window surface, then at *l* = *nl*’, this force will be greater than in the opposite case. This question, however, should be analyzed quantitatively.

It should be expected that the dynamics of the formation and collapse of the cavity should be somehow influenced by the nanobubbles of the dissolved gas. Indeed, the liquid samples were not degassed. When polymer fibers are unwound, protrusions and irregularities on the surface of the hydrophobic membrane serve as the nucleation centers of surface nanobubbles [32]. These nanobubbles are “carried” by outgrown fibers towards the cell window. The mechanical stresses resulted from abutting the unwound polymer fibers against the cell windows lead to coalescence of nanobubbles, which should contribute to the formation of the water-free cavity. This is indirectly confirmed by the results obtained with aqueous solutions of NaCl and aqueous samples subjected to shaking. However, the total number of gas nanobubbles in the volume between the polymer interface and the cell window is too small to be the key factor for the cavity formation. Indeed, as shown in References [33,34], the volume number density of nanobubbles in deionized water *n*~10^6^ cm^−3^, i.e., there exists ~10^3^ nanobubbles between the membrane surface with an area of 1 cm^2^ and the cell window. It is then clear that nanobubbles can only slightly affect the collapse rate of the cavity, but not its formation. Obviously, for a more detailed analysis, it is necessary to carry out experiments with degassed water. We, however, do not know yet how to carry out the experiments with degassed samples. The fact is that our cell was not specially hermetically sealed, and therefore the access of air during the measurements (that is why the resulting cavity collapsed) was assumed. Since the experiment lasts several hours, and the thickness of the water layer is only about 200 microns, then in a few hours the degassed sample will be completely saturated with dissolved air. Therefore, the measurements in this case will not be stationary.

Finally, the origins of the isotope effect D_2_O/H_2_O are a complete mystery with obviously huge implications that we hope to follow up. Concluding this section, we note that the model of “unwound” fibers is an alternative for the interpretation of the occurrence of the EZ near Nafion, proposed in Reference [21]. As was shown in this work, the EZ is formed only for negatively charged colloidal micron-sized particles, while for positively charged ones the effect is absent. As is known [9], the surface of Nafion in water is negatively charged due to the dissociation of terminal sulfonic groups and moving of H^+^ ions to the bulk of water. Thus the surface of unwound polymer fibers should also be negatively charged, and negatively charged particles would effectively repel from the “brush” of negative polymer fibers, while positively charged particles would attract to this brush.

In conclusion, we would like to formulate what is the novelty of this work. In our previous work [24], we showed that when a Nafion plate is immersed in water, Nafion’s fibers unwind into the water bulk, and the size of the area occupied by unwound fibers is about the EZ. It has also been shown that the effect of outgrowth is controlled by the deuterium content of liquid sample. However, in Reference [24], we could not assert with confidence that the EZ formation (pushing out of colloidal microparticles from the Nafion–water interface) is precisely due to the effect of Nafion’s fiber growth. In the present work, it was found that when the Nafion plate is immersed in a cell of limited volume, a cavity, free of water molecules, is formed almost instantly. Thus, in our experiments, the presence of colloidal microparticles is not required to prove the peculiar features of water near the Nafion surface: The water molecules themselves are pushed out. In the present work we claim that the EZ is formed as a result of the elastic properties of Nafion’s fibers, unwound in the bulk of water. These elastic properties are due to the non-additive forces of interaction between polymer fibers; these forces are described in References [43,44]. In the present work, we also qualitatively describe the role of the effect of outgrowth of polymer fibers on the transition from the hydrophobic to the hydrophilic state (an abrupt decrease in the wetting angle). Since the unwound polymer fibers prevent the contact of water molecules and the Nafion surface (water molecules reach the membrane surface, overcoming a certain resistance caused by the “brush” of the unwound polymer fibers), the transition to the hydrophilic state occurs the later, the more the fibers are unwound (see the graph in Figure 8), where, by our model, the transition to the hydrophilic state corresponds to the second jump in the dependence of 〈*C_w_*(*t*)〉. Finally, a new result is the presence of a special size *l* = (*L* − *L*_0_)/2 = 12.5 μm for Nafion N117; in this case, the growth of 〈*C_w_*(*t*)〉 upon collapse of the cavity meets a linear law with a high accuracy, and the collapse does not complete at times of ~100 min. In this case, deviations from the straight-line dependence of 〈*C_w_*(*t*)〉 are due to external impurities, e.g., an ionic component (see Figure 12), as well as preliminary treatment of the liquid, e.g., shaking (see Figure 13). We associate these features with the presence of nanobubbles, the volume number density of which depends both on the ion content and on vigorous shaking. Thus, a new result is very high sensitivity of the 〈*C_w_*(*t*)〉 dependence to external factors. For example, with NaCl additions, deviations from the straight-line dependence for 〈*C_w_*(*t*)〉 are observed up to ionic concentrations at the level of 10^−14^ M (see Figure 12).

The essential novelty in this work is the recognition of new long range non-additive forces peculiar to long thin conducting polymers (including DNA). These long ranged forces that couple these conducting polymers are not peculiar to Nafion; they are shared by interacting self-assembling DNA strands. Likewise, the Nafion-like properties should be shared with the glycocalyx of living tissue underpinning the endothelial surface layer. It is probably the reason acupuncture works. The same is true for the exothelial layers of electric fish, eels, platuses and other creatures that use electric shocks to stun prey.

## 5. Conclusions

When Nafion’s membrane swells in a cell, the volume of which is comparable to the volume of the membrane itself, various dynamic modes are realized due to the effect of “unwinding” of polymer fibers into the surrounding liquid, and they are constrained by the cell windows. If the thickness of the cell (the distance between the windows) is much less than the size of the area of ”unwinding” of polymer fibers found with fluorescence spectroscopy [24] (in this experiment, unwinding occurs in a volume that significantly exceeds the volume of the polymer plate), then the initially hydrophobic polymer fibers inevitably abut against the windows of the cell, which should lead to the appearance of a field mechanical stresses and deformations between the cell windows and the membrane interface. It is important that these stresses arise in the system of “twisted” polymer fibers, which initially has hydrophobic properties, i.e., the position of the water molecule caught in the gaps between such fibers appears to be unstable. Due to the enhancement of the hydrophobic effect caused by local stresses, the water molecules located between the polymer fibers are pushed out, which leads to the formation of an empty cavity between the membrane surface and the cell window.
